# The Atmospheric Environment Effects of the COVID-19 Pandemic: A Metrological Study

**DOI:** 10.3390/ijerph191711111

**Published:** 2022-09-05

**Authors:** Zhong Chen, Dongping Shi

**Affiliations:** 1 College of Environment and Resources, Xiangtan University, Xiangtan 411105, China; 2 Key Laboratory of Large Structure Health Monitoring and Control, Shijiazhuang 050043, China

**Keywords:** COVID-19, metrology, data analysis, atmospheric environment, pollution

## Abstract

Since the COVID-19 outbreak, the scientific community has been trying to clarify various problems, such as the mechanism of virus transmission, environmental impact, and socio-economic impact. The spread of COVID-19 in the atmospheric environment is variable and uncertain, potentially resulting in differences in air pollution. Many scholars are striving to explore the relationship between air quality, meteorological indicators, and COVID-19 to understand the interaction between COVID-19 and the atmospheric environment. In this study, we try to summarize COVID-19 studies related to the atmospheric environment by reviewing publications since January 2020. We used metrological methods to analyze many publications in Web of Science Core Collection. To clarify the current situation, hotspots, and development trends in the field. According to the study, COVID-19 research based on the atmospheric environment has attracted global attention. COVID-19 and air quality, meteorological factors affecting the spread of COVID-19, air pollution, and human health are the main topics. Environmental variables have a certain impact on the spread of SARS-CoV-2, and the prevalence of COVID-19 has improved the atmospheric environment to some extent. The findings of this study will aid scholars to understand the current situation in this field and provide guidance for future research.

## 1. Introduction

The COVID-19 pandemic caused by SARS-CoV-2 has been recognized as a global public health emergency by The World Health Organization [1]. The outbreak and continued prevalence of COVID-19 has had a severe impact on all aspects of human life worldwide. In response to the strong infectivity and spread of COVID-19, many countries have adopted policies of lockdown and restriction of activities to strictly control the occurrence of the infection. Lockdown is the most direct way to stop COVID-19, but it cannot be sustained in the context of a global pandemic. Research into specific drugs to prevent or treat COVID-19 have yet to yield results [2]. Vaccination is the most effective and cost-effective intervention to control the spread of COVID-19, but the continuous emergence of new variants of SARS-CoV-2 undoubtedly poses another great challenge to vaccine development [3].

Under the current circumstances, COVID-19 is likely to coexist with humans for a long time. Its normalization will lead to a significant decrease in the mobility of the population, making a big change in the way people live and travel, and having a great impact on the functioning of society and the policies of the state. Both natural and human behavioral changes resulting from COVID-19 have direct or indirect effects on the atmospheric environment. These influences are multifaceted, with many positive and negative influences. Also, the atmospheric environment has an important relationship to the spread of COVID-19. Understanding the interrelationship between air quality, meteorological indicators, and COVID-19 is significant for saving human lives.

We need to comprehensively understand the relationship between COVID-19 and the atmospheric environment, which has attracted the attention of many authors. But there is no metrological analysis type of review study in this field. Metrological analysis overcomes the subjective factors in the traditional literature review, completely covers all literature in the selected period, avoids the loss of key literature, and can quantitatively explore the knowledge structure, research hotspots, and the latest insights in some scientific fields [4]. The metrological study of COVID-19 and the atmospheric environment simultaneously, aiming to present the overall picture of this field by analyzing and exploring the current situation and research hotspots in this field [5].

## 2. Research Methodology

This section details the process of research design (Section 2.1), data collection (Section 2.2), and data processing (Section 2.3) used in this research.

### 2.1. Research Design

**Figure 1:**
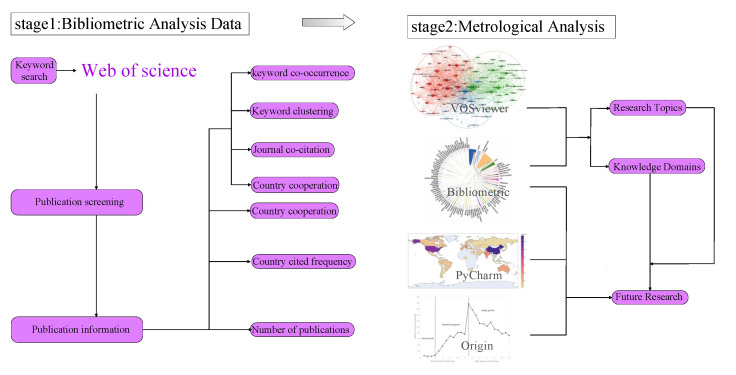
Workflow of the systematic.

The bibliometric approach is good at uncovering the underlying knowledge structure in literature and integrating the visualization results for further analysis [4]. In this paper, we use bibliometric analysis methods and data visualization analysis tools to synthesize and comb the literature related to COVID-19 and the atmospheric environment, quantitatively analyze the data, and generate visualization and content analysis results. Drawing with AutoCAD as shown in Figure 1. To understand the complex relationship between COVID-19 and the atmospheric environment as well as the degree and trend of its impact on the atmospheric environment.

### 2.2. Data Collection

The data in this paper are all from Web of Science Core Collection, which was selected because it covers a wide range of outstanding publications in the entire academic field and is one of the largest databases from 1900 to the present. The database contains authors, citations, journals, and much more bibliographic information that can be used for analysis [5,6]. The World Health Organization named the new coronavirus infection pneumonia as COVID-19, and the International Committee on Classification of Viruses named the new coronavirus SARS-CoV-2. Therefore, the search strategy was set to (COVID-19) OR (SARS-CoV-2) AND (atmosphere OR air). The search record includes title, author, abstract, keywords, and references [7]. In the Web of Science Core Collection database, the Boolean operators “OR” and “AND” are used to merge the individual components of a search query.

An initial search using these keywords from the Web of Science Core Collection database for the period 1 January 2020–31 December 2021. After filtering by publication type and research direction, a total of 1781 relevant publications were obtained by removing duplicates, including 1684 papers and 144 reviews. Research interests include environmental sciences, public environmental and occupational health, meteorology and atmospheric sciences, multidisciplinary sciences, and green and sustainable technologies, which are related to the environmental field.

### 2.3. Data Processing

We analyzed and evaluated the collected data, including country, source, title, author, publication year, affiliation, document type, field of interest, keywords, citation, etc. We consider the number of published publications as a quantitative indicator of the research productivity of authors, countries, institutions, sources, etc. The bibliometric analysis in this study was conducted mainly on VOSviewer, and the co-occurrence network visualization map was constructed based on the information extracted from 1781 publications to present the publication data. VOSviewer can combine network visualization and spectral clustering to analyze the underlying knowledge structure contained in many publications [8]. In addition, the software can perform network analysis on different aspects of the data collected, and quickly process text data downloaded directly from the Web of Science site [5]. This study also utilizes the integrated development environment PyCharm, the scientific drawing software Origin, and the spreadsheet software Excel to further assist in the analysis and processing of the data.

## 3. Results of the Metrological Analysis

This section conducts a qualitative and in-depth metrological analysis of publication data from selected studies. It includes the trend of publication volume in the last two years (Section 3.1), research content of high-influence authors (Section 3.2), national cooperation relationship (Section 3.3), analysis of the number of national citations (Section 3.4), and analysis of publication sources (Section 3.5). In addition, high co-occurrence keywords are analyzed (Section 3.6) and keyword co-occurrence cluster analysis is elaborated (Section 3.7).

### 3.1. The Number of Publications in the Past Two Years

**Figure 2:**
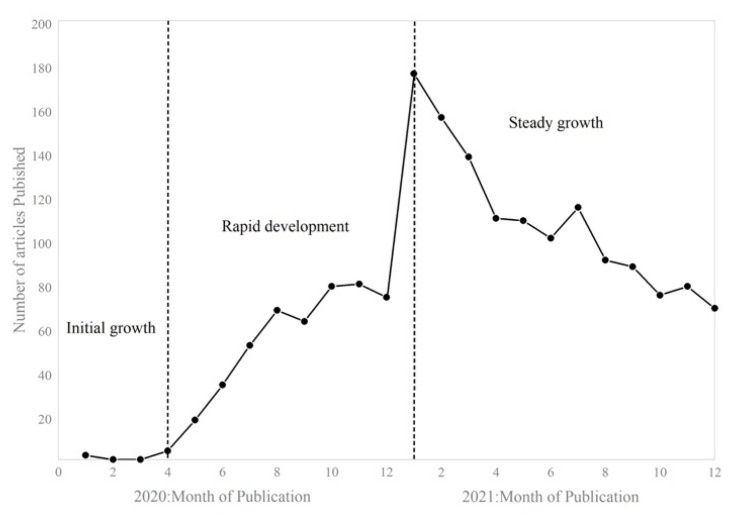
The number of publications in the past two years.

The variation in a research field’s publication output can be used to assess its development status, knowledge accumulation, and maturity [9]. As can be seen in Figure 2, the number of publications in this research area has grown rapidly over time and is divided into three phases. There were 474 publications in 2020 and 1307 publications in 2021.

The first phase is from January to March 2020, this phase is the initial growth phase. At the beginning of the COVID-19 epidemic, many countries began to focus on and study the correlation between COVID-19 and the atmospheric environment, especially the influence of the atmospheric environment on COVID-19 transmission and mortality. The first publication was published in January 2020 and focused on the extent of air and surface contamination in a COVID-19 non-intensive care unit [10]. The second publication was published in January 2020 and the study focused on the driving role of travel numbers on the spread of COVID-19 [11]. Both publications were published in Bioscience.

The second phase is from April 2020 to January 2021, and this phase is the rapid development phase. At this time, the COVID-19 outbreak has been going on for some time, research in the field expanded and the number of publications exploded. With a buffer of time and sufficient data to support it, studies on the effects of COVID-19 on air quality began to emerge and develop rapidly. The impact of COVID-19 on the atmosphere is clear and immediate. A study on NO_x_ reduction and recovery during COVID-19 in East China was published in Atmosphere in April 2020 [12]. The paper shows that NO_x_ emissions in most parts of eastern China have decreased significantly due to the lockdown after the outbreak of COVID-19. After the lockdown period, NO_x_ emissions began to rise to various degrees. Among them, January 2021 saw the maximum number of papers published, far higher than other months. The main reason may be that papers published at the beginning of the year have a longer time to accumulate citations than papers published at the end of the year, which helps to improve the impact factor.

The third phase is from February 2021 to December 2021, and this phase is the stable growth phase. The number of publications in this period began to fall from the peak, but still maintained a certain number of publications. The research focuses more on changes in air quality and emissions of atmospheric pollutants caused by COVID-19. This will be a research direction of lasting interest.

### 3.2. Influential Authors and Their Research Interests

Authors with many highly cited papers often accompany the research hotspots and methodological trends in this field and play an important role in the development of this field. In this study, the top five authors were determined by a comprehensive evaluation of the number of authors’ publications and citation times, as shown in Table 1.

Zhang, Hongliang of Fudan University published 7 papers and was cited 852 times, with an average of 121.7 times [13,14,15,16,17,18,19]. His papers were cited the most frequently and had the highest average number of citations per paper, indicating that his research received high attention from other authors. In general, the research content of these five authors has attracted much attention, and the research direction tends to be about the impact of COVID-19 lockdowns on air quality and the impact of meteorological indicators on the spread of the epidemic.

### 3.3. Cluster Analysis of National Cooperation

**Figure 3:**
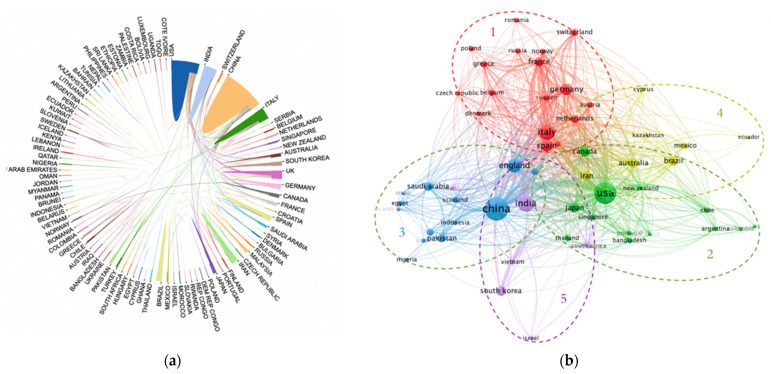
(**a**) Country partnership chart; (**b**) Country partnership clustering chart.

The number of publications is an important indicator to measure the development trend of a certain field. To some extent, the number of publications in this field can reflect the research strength of a country in this field [7]. As shown in Figure 3a, through the online bibliometric analysis website bibliometric.com (accessed on 30 April 2022), we can directly obtain the inter-country partnership map, where the area occupied by countries represents the number of national publications and the total linkage intensity represents the degree of inter-country partnership.

Figure 3b shows the graph of VOSviewer to generate the clustering analysis of country cooperation relations. In this paper, the analysis process of clustering algorithm is divided into three steps.

1. Construct an association matrix for the analysis object. The co-occurrence matrix based on the quantitative relationship between objects is a data-based square matrix. It is assumed that there are n variables in the research matrix, *x_ij_* is the co-occurrence observed value of the *i*-th variable and the *j*-th variable, and the co-occurrence observed values of all variables form a *n* × *n* square matrix [20]. The formula is as follows.
  (1) $$M=\left[\begin{array}{ccc} x_{11} & \cdots & x_{1n}\\ \vdots & \ddots & \vdots \\ x_{n1} & \cdots & x_{nn} \end{array}\right]$$
2. Calculate the similarity relation value or dissimilarity relation value (distance) between any two data objects in the matrix.
3. A certain clustering algorithm is used to divide or merge data objects to form a certain clustering result.

VOSviewer clustering algorithm can be regarded as a weighted variant based on modular clustering. The modular *Q* value calculation formula of VOSviewer is as follows, and the optimal clustering result is obtained when the modular *Q* value is the largest [20,21].

(2) $$Q=\frac{1}{2m}\Sigma_{i<j}\delta \left(x_{i},x_{j}\right)w_{ij}\left(c_{ij}-\gamma \frac{c_{i}c_{j}}{2m}\right)$$

In the above formula, m represents the total number of connections in the network; *c_ij_* represents the number of connections between nodes *i* and *j* (*c_ij_* = *c_ji_* ≥ 0); *c_i_* represents all connections of node *i*; *x_i_* represents the cluster to which the node belongs, the *δ* function is 1 for *x_i_* = *x_j_* and 0 otherwise; *w_ij_* represents the weight value *w_ij_* = 2*m*/*c_i_c_j_*, and *γ* represents the clustering parameter.

The essence of VOSviewer co-occurrence clustering is that two related items appear simultaneously and aggregate together. Different types of clustering groups can be obtained based on the measure of index clustering of the strength and direction of item correlation. VOSviewer adopts the algorithm of limiting parameter variables. By adjusting the value of limiting parameter variables, it can control small clusters, and the generated clusters have strong consistency and high stability. Using VOSviewer, the minimum number of publications is set to 7, and the minimum citation frequency is set to 10. Finally, a total of 56 countries were obtained, divided into 5 clusters. The color of the spheres represents different regions, the size of the spheres represents the number of national publications, and the total link strength indicates the intensity of cooperation between the two countries.

The first cluster (red) is dominated by Italy, which mainly cooperates closely with European countries; the second cluster (green) is dominated by the United States, which has frequent cooperation with the Americas, Oceania, Asia, Europe, and Africa; the third cluster (blue) is dominated by China, which cooperates more with European, Asian, and African countries; the fourth cluster (yellow) is dominated by Australia, which cooperates more closely with American countries; the fifth cluster (purple) is dominated by India, which cooperates more with Asian and European countries. We can find Italy, the United States, China, Australia, and India as regional leading countries, as the close cooperation between countries has played a substantial role.

The papers on COVID-19 and the atmospheric environment field are mainly concentrated in China (469), the United States (399), India (209), Italy (173), the United Kingdom (137), and other countries such as Germany (94) and Spain (89), which also have more prominent research capacity in this field. Among them, the United States is the country with the most cooperation with several countries in the world, and it frequently cooperates with China, the United Kingdom, Italy, and Canada. Globally, the international cooperation links in research in the field of the atmospheric environment under COVID-19 are strong, and many countries pay attention to research and cooperation in this field.

### 3.4. Country Cited Frequency Analysis

**Figure 4:**
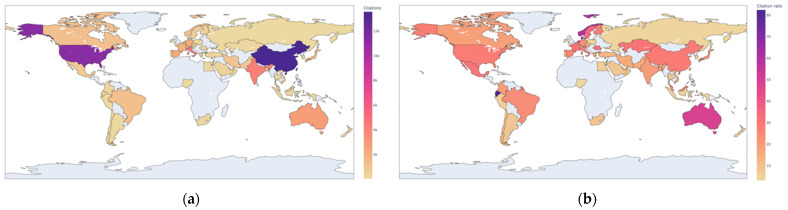
(**a**) The total number of citations by country; (**b**) The average citation rate by country.

The number of citations is an important indicator of the influence of papers in a certain field. The total number of citations and the average number of citations of a country in the field can reflect the international influence and research strength of the country in the field to a certain extent. The average number of citations can also show the quality of a country’s papers, thus reflecting the country’s scientific research capacity and level. This paper uses a simple average method. The average citation frequency is the total citation frequency divided by the total number of publications. In Figure 4a,b, the darker the color, the higher the total or the average number of citations for a country. The total number of citations in China was 13,568, with an average citation rate of 28.93. Meanwhile, the total number of citations in the United States was 11,263, with an average citation rate of 28.23. These two countries have the highest total citations, which are much higher than other countries, and the average citations are relatively good, indicating that both countries have strong research strength and influence in the field. The total number of citations in India is 4135 and the average number of citations is 19.78, which indicates that India has some research strength and influence in the field. The total citations of Australia and the United Kingdom are relatively high, with 2805 and 5781 respectively; the average citations are high, with 48.36 and 42.20, respectively, indicating the profound academic influence and first-class research level of these two countries in the field. The total number of citations in Ecuador, Norway, and Denmark is low, only 577, 1194 and 938 respectively, but the average number of citations is high, 82.43, 59.70 and 58.65, respectively, which indicates that these countries have great potential for research in this field. In contrast, it can be found that the average citation frequency data of most European countries and Australia are more prominent. This shows that the overall quality of these countries’ papers may be higher, and subject to more attention and recognition by scholars.

### 3.5. Analysis of Journal Publications and Co-Citation

A total of 137 journals have published relevant research between 2020 and 2021. Table 2 shows the information on the 10 most productive journals in this research area. In terms of the number of publications, Science of The Total Environment (203, 11.39%) is the most active journal, followed by International Journal of Environmental Research and Public Health (140, 7.86%), Environmental Research (134, 7.52%), and Aerosol and Air Quality Research (125, 7.02%). Among them, Science of The Total Environment has an impact factor of 10.753 and a citation frequency of 12,176, which is quite influential. Environmental Pollution has an impact factor of 9.988 and a citation frequency of 1644. Environmental Research has an impact factor of 8.431 and a citation frequency of 2323. They are among the most influential journals in the field of the atmospheric environment. Next, Air Quality, Atmosphere & Health has an impact factor of 5.804 and a citation frequency of 1115. Environmental Science and Pollution Research has an impact factor of 5.190 and a citation frequency of 565. These two journals are also influential in the field of the atmospheric environment.

Journal co-citation is determined based on the number of times they are cited together. Journal co-citation analysis can be used to classify journals on different topics and identify the core journals in each category. This is very helpful for authors to understand the most relevant and influential journals for a particular research topic [22]. In this paper, we use VOSviewer to perform a co-citation analysis of the collected data according to different sources, setting the minimum number of citations for a source to 200. The minimum threshold can be adjusted according to the number of publications rendered and clustering groupings. The color of the sphere represents different journal topics, and the size of the sphere indicates the number of citations. The total link strength then indicates the closeness between journals.

**Figure 5:**
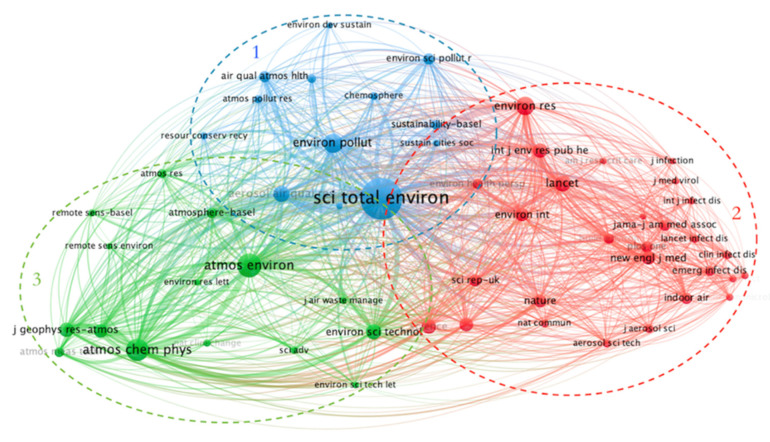
Journal co-occurrence chart.

The journals shown in Figure 5 have a significant impact on authors studying COVID-19 and the atmospheric environment. Science of the Total Environment is the most-cited journal, followed by Atmospheric Environment, Atmospheric Chemistry and Physics, Environment Pollution and Environmental research. These journals are grouped into three main groups: Environmental Science and Ecology (blue) includes science of the Total Environment, Aerosol and Air Quality Research, Environment Pollution. Environmental public health and Medicine (red) includes Environmental research, Lancet, International Journal of Environmental Research and Public Health, Science. Meteorology and Atmospheric Sciences (green) includes atmospheric Environment, Atmospheric Chemistry and Physics, Environment Science & Technology, Journal of Geophysical Research: Atmospheres. The medical journal The Lancet (202.731) and the comprehensive journal Science (63.714) have higher impact factors. The more influential journal in the environmental field is Environment Science & Technology (11.357).

### 3.6. Research Keywords

Keywords are highly generalized and concise to the subject of a paper. High-frequency keywords also reflect the research direction to some extent. The keyword density visualization can be used to quickly observe the knowledge and research density of a certain field. VOSviewer sets the minimum keyword frequency to 20 and gets a co-occurrence graph of 86 keywords after removing nonsense words. Each keyword in the figure will fill the color according to the density of its surrounding keywords. The greater the density, the closer it is to red; conversely, the smaller the density, the closer to blue. The density depends on the number and importance of keywords in the surrounding area.

**Figure 6:**
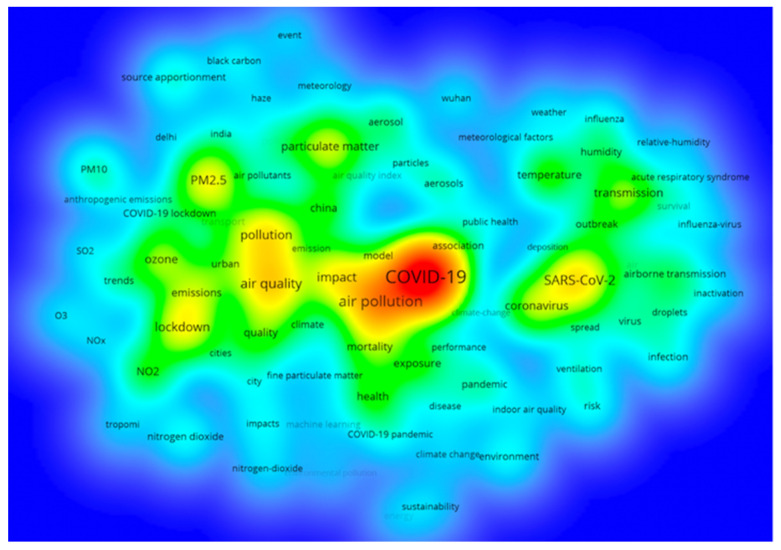
Keyword density visualization.

It can be seen from Figure 6 that in addition to air pollution and air quality, keywords related to COVID-19 mortality and environmental factors, SARS-CoV-2 and meteorological conditions also appear frequently.

### 3.7. Keyword Co-Occurrence Cluster Analysis

**Figure 7:**
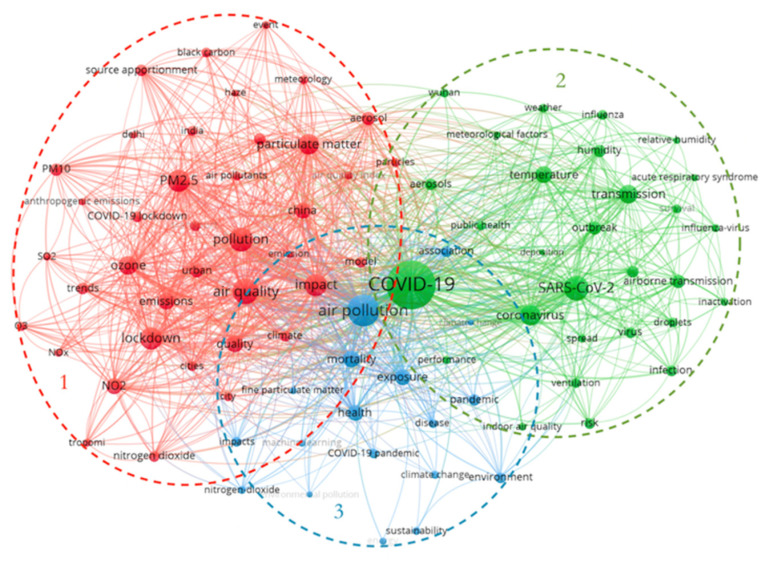
Keyword co-occurrence clustering.

VOSviewer takes a distance-based approach to visualizing bibliometric networks. After the construction of the normalized network is completed, the keywords are positioned to make the position of the strongly related keywords closer and the position of the weak related keywords further away [23]. The keyword co-occurrence networks analyze the link strength between co-occurrence keywords by studying their co-occurrence relationship in many publications. Its purpose is to describe the internal composition relationship and structure in a certain academic domain as well as to provide insights into the main research themes of the domain [24,25]. The clusters group keywords that are frequently combined in a set of keywords [26]. Different color spheres indicate different clusters, and the same sphere color represents clusters that are consistent with the research topic. The size of the spheres is proportional to the frequency of keywords. The lines between the keywords reflect the strength of their relevance [27]. A total of 5630 keywords were obtained from publications by setting the minimum keyword frequency to 20, and 86 keywords were obtained after removing meaningless words. They were divided into three major clusters, as in Figure 7.

(1) Air quality indicators (red) contain keywords “pollution”, “air quality”, “PM_2.5_”, “ozone”, “lockdown”, “NO_2_”, “particulate matter”, etc. Cluster 1 focuses on changes in the atmospheric environment and air quality in the context of COVID-19 and describes how lockdowns due to COVID-19 affect air quality.
(2) Meteorological factors affecting the spread of the outbreak (green) contain keywords “COVID-19”, “SARS-CoV-2”, “temperature”, “transmission”, “humidity”, “weather”, etc. Cluster 2 focuses on meteorological factors influencing the spread of COVID-19 and the special relationship between COVID-19 and environmental variables.
(3) Air pollution and human health (blue) contain keywords “air pollution”, “exposure”, “mortality”, “health”, “pandemic”, etc. Cluster 3 focuses on the effects of air pollution on human health and mortality.

#### 3.7.1. COVID-19 and Air Quality

The COVID-19 outbreak has had a significant impact on almost every aspect of people’s lives around the globe, resulting in a variety of direct and indirect impacts on the atmospheric environment. Shutdowns or closures of factories have reduced the amount of pollution, with an estimated 50 percent reduction in N_2_O and CO due to the closure of heavy industry during China’s lockdown [28]. Restrictions on travel, reduced mobility of people, and reduced transport and related activities have significantly reduced mobile pollution sources and pollutant emissions. PM_2.5_, PM_10_, CO_2_, and NO_2_ concentrations have all declined to various degrees during the lockdown period compared to those before the lockdown [13,29,30]. Volatile organic compounds (VOCs), including benzene, are mainly produced by vehicular traffic and other incomplete combustion processes that lockdown has increasingly limited [31]. NO_2_, one of the main indicators of world economic activity, shows signs of decline in many countries, including the United States, Canada, China, India, Italy, and Brazil [1,28,32]. At the same time, reduced activities following the lockdown have led to a broad reduction in greenhouse gas emissions. One estimate suggests that global daily CO_2_ emissions during the lockdown were 17 percent lower than the 2019 average [33]. It can be seen that the environmental conditions of various countries have changed greatly during the COVID-19 pandemic, indicating that policy intervention has played a great role.

Different studies have shown that urban lockdowns have led to considerable improvements in air quality. The positive impact of urban lockdowns on air quality is greater in cities with larger economies, more industrial activities, and higher traffic volumes [33]. According to data from the Meteorological bulletin of the atmospheric environment, the national average number of haze days in 2020 and 2021 will decrease by 1.5 and 4.4 days, respectively, compared with 2019. In 2020, the meteorological data of Shanghai during the lockdown period and before and after the lockdown were consistent. The comparison of 14 trace elements in PM_2.5_ found that the concentration of trace elements in most fine particles showed a “V” shape trend, indicating that the lockdown measures had a significant impact [34]. Comparing the air quality index (AQI) results before and after the impact of COVID-19 across India shows that most pollutant concentrations (PM_10_, PM_2.5_, CO, SO_2_, NO_x_) show different patterns of gradual to rapid decreases [35,36]. A wavelet analysis of COVID-19 confirmed data and weather data in California from 1 March to 24 May 2020 found that AQI and COVID-19 showed negative correlation circles during the observation period, suggesting that COVID-19 leads to better AQI and less environmental pollution [37].

However, with the end of the lockdown and the resumption of normal activities, pollutant emissions rebounded somewhat, and air pollution gradually returned to near pre-COVID-19 levels. NO, NO_2_, and NO_x_ all exhibit abrupt decreases at the time the United Kingdom locked down. But the return of vehicles to the road during early lockdown has already offset much of the air quality improvement seen when locked down [38]. Most of the lockdown’s impact on the atmosphere is short-term, but changes in human activities caused by the ongoing COVID-19 pandemic can also have lasting effects on the atmosphere.

COVID-19 has also had some adverse effects on the atmospheric environment, with changes in O_3_ due to changes in NO_x_ and VOC_s_ emissions. The significant reduction of NO_x_ during the lockdown was the main reason for the significant increase in O_3_. Compared with the same period of the previous year, O_3_ in most parts of the world showed various degrees of increase during the epidemic lockdown period [39,40,41]. AQI results in some parts of India comparing the 2020 lockdown period with 2019 show a sharp decrease in NO_2_ and an increase in O_3_ [13,35]. During the nationwide implementation of restrictions in China in 2020, NO_x_ generated during transportation decreased significantly (>50%), O_3_ concentration in the air increased significantly, and the atmospheric oxidation capacity (AOC) in the Yangtze River Delta region increased significantly (up to 25%), which was also a major reason for the increase of O_3_ level during city lockdown [14].

#### 3.7.2. Meteorological Factors Affecting the Spread of COVID-19

Meteorological factors are unstable and diverse, and the impact of meteorological indicators on the spread of COVID-19 is comprehensive and complex. Analysis of Canadian meteorological data and COVID-19 confirmed cases for 2020 revealed a direct negative correlation between air quality, temperature, humidity, and COVID-19 infection [42]. A study of meteorological data and COVID-19 data in Istanbul and other regions also came to the same conclusion that air quality and temperature significantly affect the number of COVID-19 deaths in Istanbul [43]. In the same analysis of Wuhan, there was also a significant agreement between AQI, humidity, and mortality rates. Humidity was negatively correlated with related COVID-19 deaths [44]. Temperature is the only significant meteorological indicator that has a significant correlation with the spread of COVID-19 [45]. Although only in the short term, daily temperatures in Madrid, Spain, and California, the United States show a negative correlation between COVID-19 outbreaks and death rates [37,46]. This means that temperature plays an important role in limiting COVID-19, suggesting that temperature may help contain COVID-19. Overall, empirical results suggest that rising temperatures may reduce transmission of the SARS-CoV-2.

Statistical analysis of confirmed COVID-19 data and local meteorological variables in Manaus revealed that low solar radiation cycles may lead to increased COVID-19 deaths due to reduced solar radiation. Dry spells may impair nasal functions that prevent viruses and bacteria from entering the body, leading to increased mortality from COVID-19 [47]. A study in Italy found that cities with high wind speeds had fewer COVID-19 infections, and inland cities with low wind speeds and high air pollution had higher COVID-19 infections [48]. Low wind speeds and high concentrations of air pollutants may contribute to the persistence of virus particles in urban air and thus to the indirect transmission of SARS-CoV-2.

#### 3.7.3. Air Pollution and Human Health

Air pollution is one of the biggest environmental threats to human health. It is harmful to the human body in many ways, mainly in the form of respiratory diseases and physiological disorders. It is of great practical significance to study the impact of air pollution on aggravating COVID-19 infection in the population. People living in areas with high levels of pollutants are more likely to develop chronic respiratory diseases and infections with pathogens [49].

An analysis of the link between air pollution and COVID-19 in Indian cities found an asymmetric relationship between PM_2.5_ and COVID-19 cases, where the positive impact of PM_2.5_ concentration intensifies the spread of COVID-19. Environmental pollutants CO, O_3_, and NO_2_ are also positively correlated with confirmed cases and deaths of COVID-19 [50]. Atmospheric particulate matter, upon exceeding the satisfactory level, serves as an important cofactor in increasing the risk of SARS-CoV-2 transmission and related mortality [51].

Data from Tehran also shows a significant link between COVID-19 mortality and exposure to environmental pollution, with increased PM_2.5_ levels in the air likely to increase SARS-CoV-2 mortality [52]. A similar study conducted in Germany showed that PM_2.5_, O_3_, and NO_2_ were significantly correlated with COVID-19 outbreaks [45]. In many Italian provinces, long-term air quality data are significantly associated with COVID-19 cases, further demonstrating that long-term exposure to air pollution may be an enabling environment for virus transmission [53]. Data from Wuhan also points to an increase in deaths due to poor air quality [44]. Environmental pollution is an important factor affecting the incidence and death of COVID-19. Areas with higher levels of environmental pollution are prone to respiratory syndrome, which reduces the immunity of residents and affects their susceptibility to COVID-19 [54]. The mortality rate decreases more significantly in countries with high levels of greenery than in countries with low levels of greenery. A good air environment and a green environment are conducive to human survival and development [55].

## 4. Conclusions

At the beginning of COVID-19, many authors began to study the meteorological factors affecting the spread of COVID-19 and the impact of air pollution on human health and mortality, which is also a continuing hot research direction. Studies have shown that good air quality, high temperature, and humidity are relatively adverse to COVID-19 infection. Dry air may impair nasal functions that prevent viruses and bacteria from entering the body, leading to increased mortality from COVID-19. Low solar radiation cycles may lead to higher COVID-19 mortality due to reduced solar radiation [47]. Rising temperatures could help curb the spread of COVID-19. Low wind speed and high concentration of air pollutants may promote the persistence of virus particles in urban air, thus facilitating the indirect transmission of SARS-CoV-2 [48]. People living in areas with high levels of pollutants are more likely to suffer from chronic respiratory diseases and to be infected with pathogens [49]. The higher the level of environmental pollution, the more likely it is to affect the susceptibility to COVID-19 [54]. Understanding SARS-CoV-2 transmission under environmental variables can help provide supporting evidence to healthcare policymakers for formulating strategies to combat COVID-19.

Subsequently, there have been more and more papers on the impact of COVID-19 on the atmospheric environment and air quality, which has become the hottest topic in the field of the atmospheric environment under the impact of COVID-19. Obtaining sufficient atmospheric environment data requires a time buffer, so papers on this topic appeared relatively late. Air pollution is already a serious environmental problem. The COVID-19 pandemic has had a great impact on the atmospheric environment and improved the atmospheric environment to a certain extent. During the COVID-19 pandemic, lockdowns, travel restrictions, and reductions in industry, transportation, and related activities have significantly reduced mobile sources of pollution and emissions of pollutants. The lockdown has also led to widespread reductions in air pollutants and greenhouse gas emissions, and considerable improvements in air quality. In general, the concentrations of the environmental pollutants PM_2.5_, PM_10_, CO_2_, CO, SO_2_, and NO_2_ all declined to various degrees, while the concentration of O_3_ showed an increasing trend. Studying the special and complex relationship between COVID-19 and the atmospheric environment can provide new insights for environmental managers to control good air quality and manage air pollution under the impact of COVID-19.

## Figures and Tables

**Table 1:** Top 5 most productive authors on COVID-19 and air environment research.

Rank	Author	Institution	TP	TC	AC	Main Research Interests
1	Zhang, Hongliang	Fudan University	7	852	121.7	The impact of lockdown measures on air quality in China and India
2	Coccia, Mario	CNR Natl Res Council Italy	6	558	93	The impact of air pollution on the spread of outbreaks
3	Bashir, Muhammad Farhan	Cent South University	6	539	89.8	Environmental quality, climate indicators and the COVID-19 pandemic
4	Querol, Xavier	Spanish Res Council CSIC	5	423	84.6	Changes in air quality caused by COVID-19 lockdown and its implications
5	Wang, Peng	Hong Kong Polytech University	7	358	51.1	Changes and causes of air pollutant concentration under epidemic lockdown

Notes: TP = Total publications; TC = Total citations; AC = Average number of citations per paper.

**Table 2:** The top 10 most active journals in terms of publication.

Rank	Journal Title	TP	TP (%)	TC	IF (2021)	Subject Category of the Journal
1	Science of The Total Environment	203	11.39%	12,176	10.753	Environmental Sciences
2	International Journal of Environmental Research and Public Health	140	7.86%	1368	4.614	Public Environmental & Occupational Health
3	Environmental Research	134	7.52%	2323	8.431	Environmental Sciences
4	Aerosol and Air Quality Research	125	7.02%	1384	4.53	Environmental Sciences
5	Sustainability	109	6.12%	542	3.889	Green & Sustainable Science & technology
6	Environmental Science and Pollution Research	89	5.00%	565	5.190	Environmental Sciences
7	Atmosphere	81	4.55%	466	3.110	Meteorology & Atmospheric Sciences
8	Scientific Reports	75	4.21%	832	4.996	Multidisciplinary Sciences
9	Environmental Pollution	60	3.37%	1644	9.988	Environmental Sciences
10	Air Quality Atmosphere and Health	55	3.09%	1115	5.804	Environmental Sciences

Notes: TP = Total publications, TP (%) = Total publications (%), IF (2021) = Impact factor in 2021, TC = Total citations.

## Data Availability

Not applicable.
